# Preparation of a Solvent-Resistant Nanofiltration Membrane of Liquefied Walnut Shell Modified by Ethylenediamine

**DOI:** 10.3390/membranes13080719

**Published:** 2023-08-04

**Authors:** Ayang Zhou, Mingxue Cao, Demeng Qian, Jingyao Zhang, Yaping Sun

**Affiliations:** 1 School of Materials and Chemical Engineering, Chuzhou University, Chuzhou 239000, China; mingxuecao@outlook.com (M.C.); syp04hd@163.com (Y.S.); 2 School of New Energy and Intelligent Connected Vehicles, Anhui University of Science and Technology, Huainan 232000, China; 3 College of Chemical and Materials Engineering, Xuchang University, Xuchang 461000, China; 18738616818@163.com

**Keywords:** DMF, ethylenediamine, liquefied walnut shells, nanofiltration, modified

## Abstract

N,N-dimethylformamide (DMF) has excellent chemical stability and is widely used as an aprotic polar solvent. In order to reduce production costs and reduce pollution to the surrounding environment, it is necessary to recycle and reuse DMF. Previous research has found that the thin film composite nanofiltration membrane prepared from liquefied walnut shells exhibited a high rejection rate in DMF, but relatively low permeance and mechanical strength. In order to increase permeance without compromising the separation performance, ethylenediamine (EDA) is used as a modifier to graft onto the structure of liquefied walnut shell through the Mannich reaction. Then, modified liquefied walnut shell as an aqueous monomer reacts with trimesoyl chloride (TMC) via the interfacial polymerization method on the EDA-crosslinked polyetherimide (PEI) membrane. The results show that the permeance of the prepared membrane is significantly improved by an order of magnitude, demonstrating a rejection rate of 98% for crystal violet (CV), and a permeance of 3.53 L m^−2^ h^−1^ bar^−1^ in DMF. In conclusion, this study reveals the potential of utilizing liquefied walnut shells as raw materials for preparing high-performance separation membranes and demonstrates that surface modification is a feasible approach to enhance permeance of membranes without sacrificing the rejection rate.

## 1. Introduction

DMF is a widely used solvent in the chemical and textile industries. It is an ideal industrial solvent due to its excellent solubility and stability [1,2,3]. DMF is currently widely used in the production of synthetic fibers, plastics, rubber, coatings, disinfectants, and pharmaceuticals, among other fields. However, during industrial production, DMF generates a large amount of waste liquid, which poses certain environmental hazards [3,4,5,6]. Waste liquid recycling is an important method for solving the problem of DMF waste liquid. Currently, many enterprises have begun to adopt scientifically effective waste liquid recycling processing methods, such as bio-treatment technology and physical and chemical treatment technology [7,8,9]. These technologies can effectively recover DMF, reducing pollution in the environment.

Compared to traditional water treatment technologies, membrane technology is characterized by its simplicity, efficiency, energy conservation, environmental protection, and high effluent quantity [10,11,12]. It has a series of outstanding advantages such as its mild operating conditions, small footprint, and being easy to scale up and maintain, etc., in the field of water treatment. It thus occupies an important position as one of the most rapidly developing technologies in the chemical industry in recent years. Currently, membrane technology has been used for the removal of suspended solids, bacteria, viruses, colloids, hardness, heavy metals, and dissolved organic matter [13,14,15,16,17]. In recent years, with the innovation of membrane preparation methods and analytical methods, as well as the rapid development of material chemistry, more and more advanced separation membranes have emerged, aiming to further improve membrane performance, while reducing energy consumption and costs. The demand for membranes with high performance in durability, selectivity, and flux has been a challenging task for researchers in the field. Among the various types of NF membranes, solvent-resistant NF membranes have attracted significant attention due to their resistance to organic solvents and high selectivity for separations.

Thin film composite material (TFC) membrane is composed of a support layer and a top layer, which allows solvent molecules to penetrate and intercept solutes. The support layer has a certain mechanical strength, and the ultra-thin top layer plays a crucial role in its separation performance. Research has found that the performance of TFC membranes can be improved by optimizing the preparation conditions of the membrane, such as optimizing the concentration and type of reaction monomers, polymerization conditions, heat treatment process, and crosslinking, etc. [8,18,19,20]. Polyamide-based membranes are the most widely used solvent-resistant NF membranes due to their high selectivity and excellent solvent resistance properties. Nevertheless, several barriers exist to their application, such as low water permeability, low flux, and poor mechanical properties. To overcome these limitations, recent studies have focused on the modification of polyamide-based NF membranes by doping with metal–organic framework (MOF) materials, such as zeolitic imidazole framework (ZIF), covalent organic framework (COF) and 2D graphene-based materials [21]. This approach aims to enhance the mechanical and separation properties of the membranes while maintaining their solvent-resistant properties. Another strategy to improve the performance of polyamide-based NF membranes is to develop ultra-thin skin layers with high water permeability. Huang et al. prepared the OSN membranes by simultaneously reducing the thickness of the thin film layers (<10 nm) and introducing permanent intrinsic porosity within the membrane (6.3 Å), and the membranes had a high rejection rate (>95%) for dyes with MWCO of 450 Da [22]. Although this approach has demonstrated excellent flux performance, it often results in a reduction in the mechanical properties of the membranes, reducing their durability and lifetime. For instance, Li et al. prepared ultra-thin OSN nanofilms isolated and supported by a wire loop with a molecular weight cut-off of 233 Da and MeOH permeance of 13 LMH/bar [23]. Wan et al. prepared a thin film composite (TFC) membranes through cross-linking of β-cyclodextrin and piperazine layers using the trimesoyl chloride vapor method, and the methyl blue (MB, MW = 800 Da) rejection rate and ethanol permeance reached 96.6% and 12.5 LMH/bar, respectively [24].

Polyphenols have multiple reactive groups in their molecular structure, which can be used for etherification, acylation, electrophilic substitution, phenol condensation and the Mannich reaction [25]. Walnuts belong to the genus *Juglans*, and the *Juglans regia* family, and are widely distributed around the world. Walnut shells account for about 30% of the walnut mass. After processing, walnuts produce a large amount of walnut shells. Most walnut shells are currently subjected to burning treatment, causing a waste of resources and environmental pollution [26]. In recent years, much attention has been given to polyarylester membranes derived from polyphenols, such as catechol, gallic acid, and tannic acid [27,28,29]. These prepared nanofiltration membranes exhibited favorable application for the treatment of dye wastewater and organic solvent recovery.

**Scheme 1:**
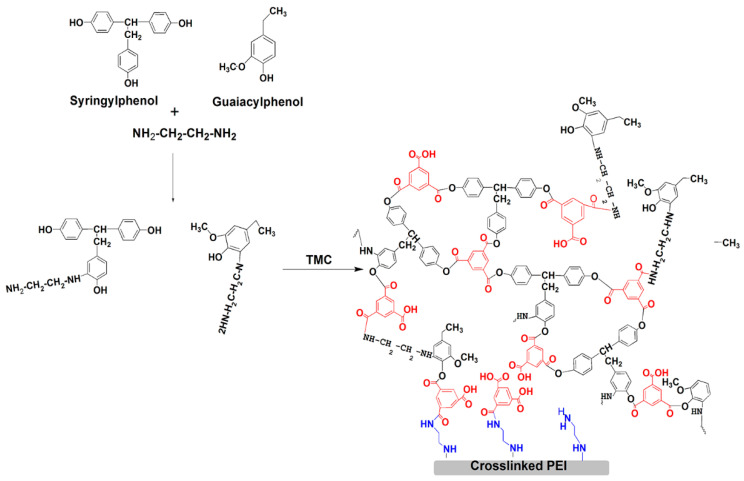
A possible reaction mechanism of solvent-resistant composite membrane prepared via the reaction of liquefied walnut shell modified by EDA with pyromellitic chloride on a crosslinked PEI support.

In previous studies, we prepared liquefied walnut shell polyarylester (LWP) membranes and explored their separation performance and application in potential applications in DMF treatment. The optimal NF-2LWP membrane maintained a stable DMF permeance of 0.22 L m^−2^ h^−1^ bar^−1^ and a rejection rate of 98% for crystal violet (CV, 407.98 g mol^−1^). The separation efficiency of the nanofiltration membrane prepared using liquefied walnut shells as raw materials was promising; however, its permeance was relatively low, along with its mechanical strength [30]. Hydrophilicity is important for solute removal from organic solvents; for instance, Van der Bruggen studied the wetting effects on the separation performance, indicating that apolar solvents lead to lower permeance when using hydrophilic membranes but higher permeance when using hydrophobic membranes. They attributed this phenomenon to the effect of the surface tension of solvents on the membrane [31]. In particular, D. Bhanushali et al. demonstrated that the permeability of hydrophilic membranes in polar solvents is 8–10 times higher than in hydrophobic membranes, while the permeability of hydrophobic membranes in non-polar solvents is 2–4 times higher than that in polar solvents [32]. In order to increase flux without reducing rejection performance, the liquefied walnut shells were subsequently subjected to modified treatment. In order to increase the flux of the membrane, a Mannich reaction process was used to graft hydrophilic EDA onto the structure of the liquefied walnut shells. As shown in Scheme 1, the liquefied walnut shell with abundant Guaiacol and Syringol structural units, after being modified by EDA, undergoes interfacial polymerization with TMC, and forms a relatively dense solvent-resistant composite film on the crosslinked polyetherimide surface. The resulting surface-grafted EDA chains endowed the resulting membranes with improved hydrophilicity and mechanical strength, as indicated by contact angle measurements and nanoindentation testing tests. A variety of characterization techniques including Poisson testing, atomic force microscopy, and scanning electron microscopy were used to analyze the prepared membrane, and to further investigate its separating performance. The results show that the prepared membrane material exhibited a significant increase in permeance by one order of magnitude while minimizing the reduction in the rejection rate. Overall, this study reveals the potential of using liquefied walnut shells as raw materials for the preparation of high-performance separation membranes, and suggests that surface modification treatment is a viable method for improving flux performance without sacrificing the rejection rate.

## 2. Materials and Methods

### 2.1. Materials

Walnut shells were obtained from Yangbi Yi Autonomous County, Yunnan Province, China. The non-woven fabric material was polypropylene, and the manufacturer was Tianjin Teda filters Co., Ltd. (Tianjin, China). Polyetherimide (PEI, Ultem 1000) was purchased from Saudi Basic Industries Corporation (Riyadh, Saudi Arabia) as a base membrane material. Crystal violet (CV, 407.98 g mol^−1^) and ethylenediamine were supplied by Chuangshi Chemical Co., Ltd. (Jinan, China). Dimethylacetamide (DMAc) was supplied by MYM Biotechnology Co., Ltd. (Shanghai, China). Trimesoyl chloride was supplied by HEOWNS (Tianjin, China), and was used as a monomer for interfacial polymerization. N,N-dimethylformamide (DMF) was purchased from Kemiou Chemical Reagent Co., Ltd. (Tianjin, China). All reagents were not purified further.

### 2.2. Membrane Preparation

#### 2.2.1. Preparation of Polyether-Imide Support Membrane and Crosslinked PEI Membrane by EDA

We dissolved polyether imide in DMAc for 4 h, and then, after vacuum deaeration, scraped polyether imide on non-woven fabric with a calibrated scraper to control the scraping membrane thickness to 150 μm to obtain a polyether imide support membrane. The polyether Imide base membrane was fixed in a polytetrafluoroethylene frame, and then 100 mL of ethylenediamine methanol solution with a mass–volume ratio of 6:100 was poured onto the surface of the membrane for cross-linking. The cross-linking time was 1 h, followed by removing the excess solution, and the membrane was stored in a refrigerator at 8 °C.

#### 2.2.2. Preparation of EDA-Modified TFC Membranes

The preparation details of liquefied walnut liquid can be found in the previously published literature [30]. After the reaction of 2 g of liquefied walnut solution with 1 g of EDA for 1 h, the solution was poured onto the surface of EDA-crosslinked PEI. After two minutes, the excess liquid on the surface was wiped off and a TMC n-hexane solution with a mass–volume ratio of 0.2:100 was poured onto the membrane surface for 1 min. Then, the membrane was put into a microwave oven for heat treatment with the frequency of 2450 MHz for 1 min, the surface was rinsed with deionized water, and then was placed in the refrigerator for testing The cross-linked membrane was named C-PEI. NF-E-xLWP was used to represent the prepared composite membrane, where x represents the grams of liquefied walnut shells used. The composite membrane without EDA modification was named NF-xLWP. At the same time, the membrane of cross-linked polyether imide directly introduced into TMC was named NF-C for comparison.

### 2.3. Membrane Structural Characterization

To further characterize the membrane material, a series of detailed characterization tests were conducted. ATR total reflection Fourier transform infrared spectroscopy (ATR-FTIR) is a non-destructive analytical technique that does not require sample preparation and is used to analyze the chemical structure of membrane surfaces (Thermo Scientific, NICOLET 6700, Waltham, MA, USA). Poisson testing was used to determine the membrane’s elastic modulus and Poisson’s ratio. (Lisheng, LS1000, Jinan, China). Atomic force microscopy was employed to observe the morphology of the membrane surface (Seiko, SPA400, Tokyo Metropolitan, Japan), while nanoindentation testing was utilized to investigate the mechanical properties of the membrane material (Felles, FPNAN-3Dcom, Shanghai, China). Scanning electron microscopy was used to analyze the structure of the membrane before and after undergoing the modified treatment (Zeiss, sigma300, Obercohen, Germany). X-ray photoelectron spectroscopy (XPS) is a technique for analyzing the surface chemical properties of materials. XPS was used to measure the elemental composition and chemical valence states of the membrane surface (Thermo Scientific ESCALAB 250XI, Waltham, MA, USA).

## 3. Results

**Figure 1:**
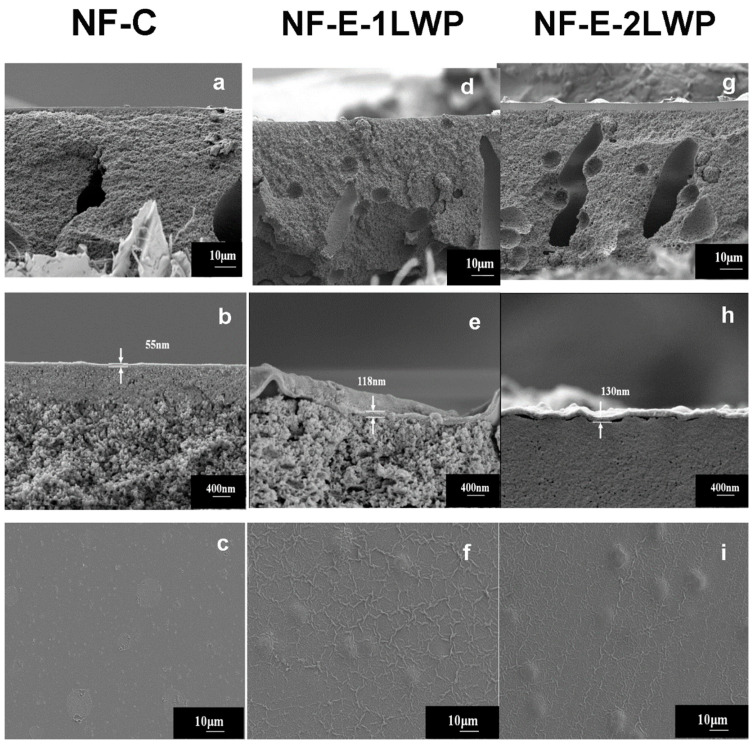
SEM images of NF-C (**a**–**c**), NF-E-1LWP (**d**–**f**) and NF-E-2LWP (**g**–**i**).

The SEM images of the prepared membrane are shown in Figure 1. The surface of the membrane prepared by directly pouring TMC onto the cross-linked polyetherimide membrane has a relatively thin top layer. Compared with the membrane prepared via the reaction of TMC, the thickness of the membrane modified with EDA is thicker. The thickness of the membrane increases from 118 nm for NF-E-1LWP membrane to 130 nm for NF-E-2LWP membrane. From the subsequent results of XPS spectra, the amount of ester groups on the membrane surfaces decreases in the order of NF-E-2LWP, NF-E-1LWP and NF-C, which results in a higher thickness of the top layer.

**Figure 2:**
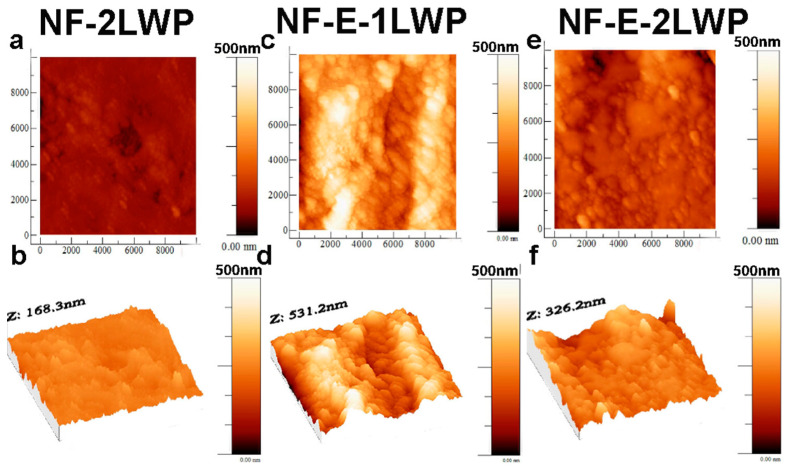
AFM images of NF-2LWP (**a**,**b**), NF-E-LWP (**c**,**d**) and NF-E-LWP (**e**,**f**).

The AFM image of the prepared membrane is shown in Figure 2. The average roughness of several membranes including NF-2LWP, NF-E-LWP and NF-E-2LWP is 14.38 nm, 82.26 nm and 39.96 nm, respectively, indicating that the modifications with EDA visibly change the morphology of the LWP-TMC membrane. Compared with NF-2LWP, the relative roughness of the modified membrne is higher, which may be due to the interaction between EDA and liquefied walnut shells.

**Figure 3:**
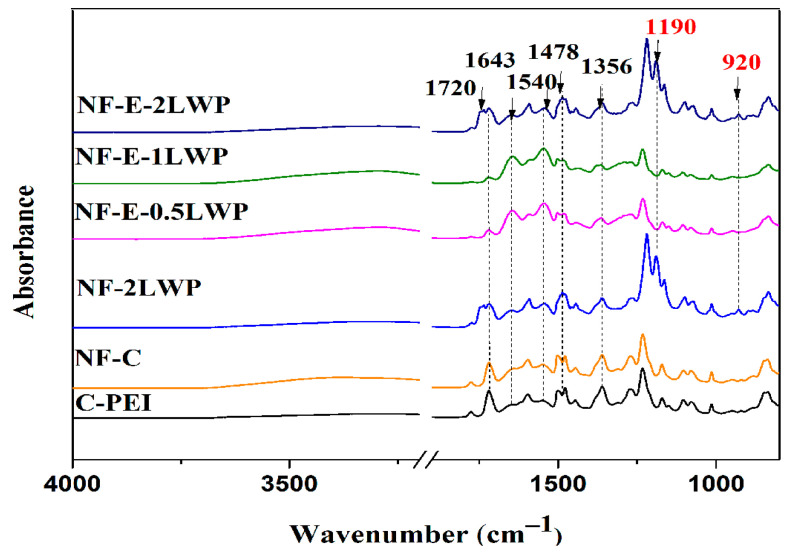
ATR-FTIR spectra of C-PEI, NF-C, NF-2LWP, NF-E-0.5LWP, NF-E-1LWP and NF-E-2LWP membranes.

ATR-FTIR spectroscopy was employed to analyze the chemical structure of the membrane surface. As shown in Figure 3, the absorption peaks at 1720 and 1356 cm^−1^ are characteristic peaks of the imide group of the PEI membrane; the characteristic absorption peaks of the amide group of the EDA-crosslinked PEI membrane appear at 1643 cm^−1^ and 1543 cm^−1^ due to the transformation of some imide groups to amide groups [33]. After interfacial polymerization, the intensity of the absorption peaks at 1650 cm^−1^ and 1543 cm^−1^ of the NF-C membrane is higher than that of the C-PEI membrane, indicating the formation of a new polyamide layer on the surface of TMC and C-PEI. For the nanofiltration membrane produced by means of interfacial polymerization with liquefied walnut shells, a characteristic absorption peak of the C-O-C bond appears at 1190 cm^−1^, and a characteristic absorption peak of the carboxyl group (-COOH) appears at 920 cm^−1^, in addition to the characteristic peaks of the amide group at 1643 cm^−1^ and 1540 cm^−1^ [34]. It can be seen that the ester group peak intensity at 1190 cm^−1^ is higher for NF-2LWP and NF-E-2LWP than for NF-E-0.5LWP, indicating that the intensity of the ester group at 1190 cm^−1^ gradually increases with the increase in liquefied walnut shell concentration.

**Figure 4:**
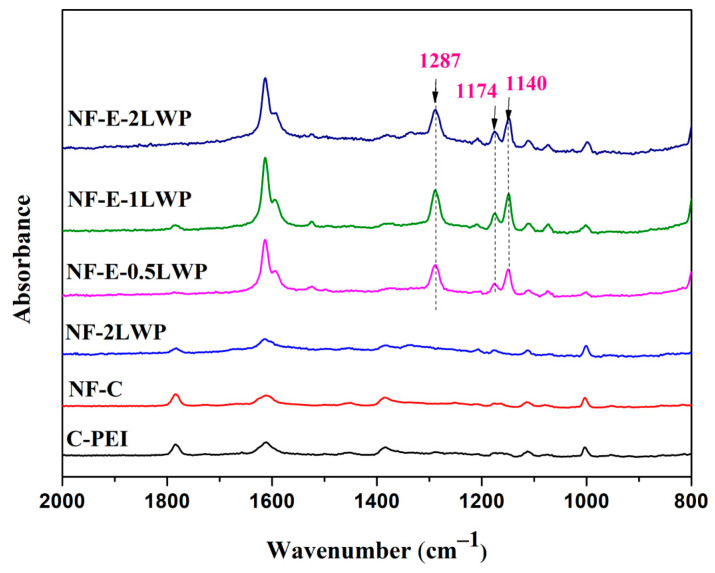
Raman spectra of C-PEI, NF-C, NF-2LWP, NF-E-0.5LWP, NF-E-1LWP and NF-E-2LWP membranes.

As shown in Figure 4, the N-H out-of-plane deformation vibration corresponding to the benzene ring at 1287 cm^−1^ and the C-N vibrations corresponding to at 1287 cm^−1^ and 1145 cm^−1^ indicate that liquefied walnut shell can undergo the Michael addition reaction with ethylenediamine, and the amine group of ethylenediamine is grafted onto the benzene ring of liquefied walnut shell.

**Figure 5:**
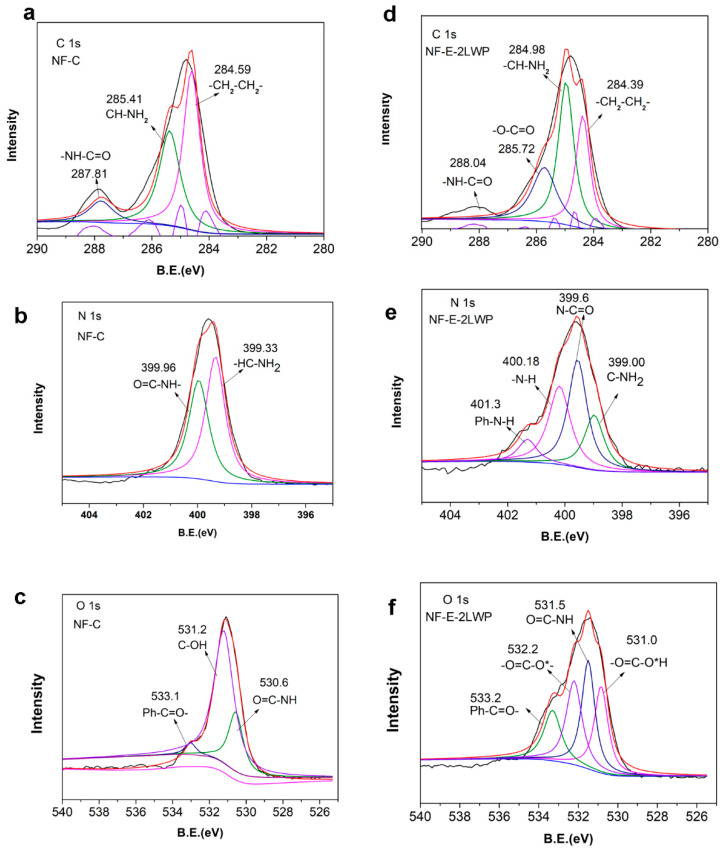
Deconvoluted C1s, N1s and O1s XPS spectra of NF-C (**a**–**c**) and NF-E-2LWP membranes (**d**–**f**).

Figure 5 shows the XPS spectra of carbon, nitrogen, and oxygen in NF-C and NF-E-2LWP: for NF-C, the carbon characteristic peaks are located at 284.59 ev (-CH_2_-CH_2_-), 284.9 ev (C=O), and 285.41 ev (C-N), respectively. The nitrogen characteristic peaks are located at 399.33 ev (-C-NH_2_) and 399.96 ev (-HN-C=O). The oxygen characteristic peaks are located at 530.6 ev (-HN-C=O), 531.2 ev (C-OH) and 533.1 ev (Ph-C=O-). For NF-E-2LWP, the carbon characteristic peaks are located at 284.39 ev (-CH_2_-CH_2_-), 284.98 ev (C-N), 285.72 ev (-O-C=O) and 288.04 ev (-NH-C=O). The nitrogen characteristic peaks are located at 399.00 ev (-C-NH_2_), 399.60 ev (-HN-C=O), 400.18 ev (-NH), and 401.3 ev (Ph-NH-). The oxygen characteristic peaks are located at 531.50 ev (-HN-C=O), 532.20 ev (-O*-C=O), 533.20 ev (Ph-C=O) and 531.00 ev (C-OH) [35]. The appearance of ester groups proves that the phenolic hydroxyl group of liquefied walnut shell reacts with TMC. The appearance of amine groups connected to the benzene ring (Ph-NH-, 401.3 ev) also proves that EDA is grafted onto liquefied walnut shells. Table S1 displays the C, N and O compositions of the C-PEI, NF-C, NF-2LWP and NF-E-2LWP membranes. By comparing the measured O/N value of the membrane’s top layer, it can be found that the O/N ratio of NF-E-1LWP and NF-E-2LWP is 0.64 and 0.69, respectively; as the concentration of liquefied walnut shell increases, the O/N value of becomes higher, which is attributed to the formation of more ester groups via the interfacial polymerization reaction.

**Figure 6:**
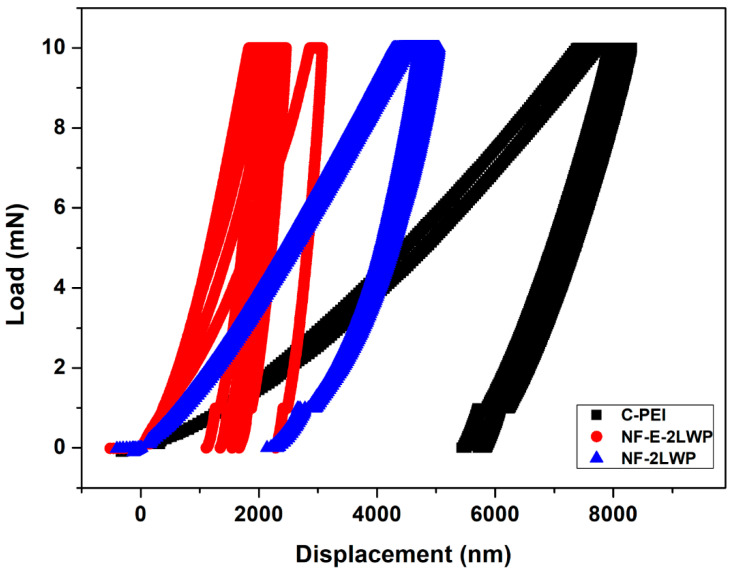
Load–displacement curves of three composite nanofiltration membranes, including C-PEI, NF-2LWP, and NF-E-2LWP.

The mechanical properties of C-PEI, NF-E-2LWP and NF-2LWP membranes were compared via nanoindentation technology, and the effects of interfacial polymerization on the mechanical properties of nanofiltration membranes were studied. The nanoindentation method is a commonly used method to characterize the mechanical properties of thin films at the nanoscale. During the experiment, the indenter continued to press into the surface of the membrane at a depth of approximately 500 nm, during which both elastic and plastic deformation generally occurred simultaneously. Subsequently, the load was slowly removed to obtain an unloading curve for elastic displacement recovery. Figure 6 shows the load–displacement curve of the membrane surface during a cyclic loading and unloading process. In the initial stage of 50–100 nm, the load of the NF-E-2LWP membrane gradually exceeds that of the other two membranes. This depth range corresponds to the thickness of the active layer of the nanofiltration membrane, and the curves within this range reflect the mechanical properties of the nanofiltration membrane’s top layer. The load on the membrane is in the order of NF-E-2LWP > NF-2LWP > C-PEI membrane. On the basis of the original cross-linked membrane, interface polymerization can help to improve the mechanical strength of the membrane. Compared with NF-2LWP, the addition of EDA during the interface process increases the cross-linking degree of the top layer of NF-E-2LWP, resulting in an overall increase in the mechanical strength of the membrane.

Poisson’s ratio is a parameter used to measure the fluid properties in the physical properties of the medium, representing the ratio of the space occupied by the liquid per unit volume to the space occupied by the liquid itself. Table 1 displays the Poisson’s ratios of the C-PEI, NF-2LWP and NF-E-2LWP membranes. Compared with NF-2LWP and NF-E-2LWP, C-PEI has a small Poisson’s ratio of 0.1, which means that it has good viscosity and flow performance. With the occurrence of interfacial polymerization, the density of the membrane increases, the Poisson’s ratio increases, and the fluidity decreases.

**Figure 7:**
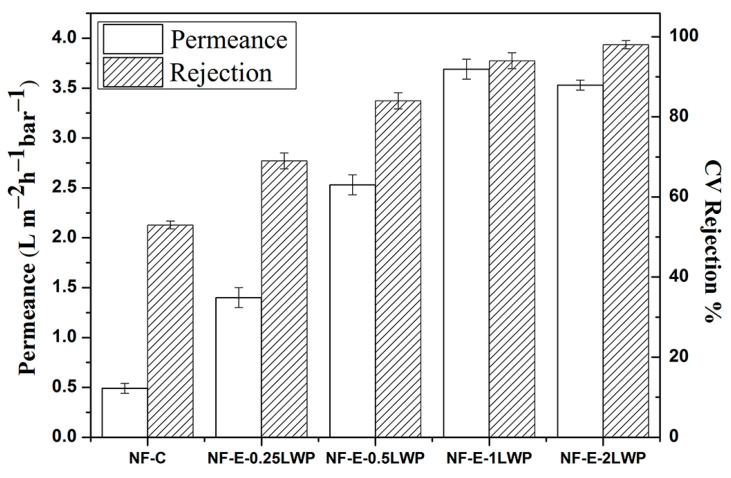
The effects of LWP concentration on the performance of membranes in DMF.

For NF-C, due to the absence of LWP, the rejection rate of the membrane is relatively low at around 53%. As the concentration of liquefied walnut shell increases, the rejection rate of the membrane also increases, as shown in Figure 7. When the liquefied walnut shell concentration reaches 2 g, the rejection of the membrane reaches 98%, and the permeance increases. When the concentration of liquefied walnut shell reaches 2 g, compared to 1 g, the permeance slightly decreases to 3.53 L m^−2^ h^−1^ bar^−1^, which may be due to the increase in the thickness of the top layer increasing the mass transfer resistance of the membrane as shown in the scanning electron microscopy in Figure 1.

**Figure 8:**
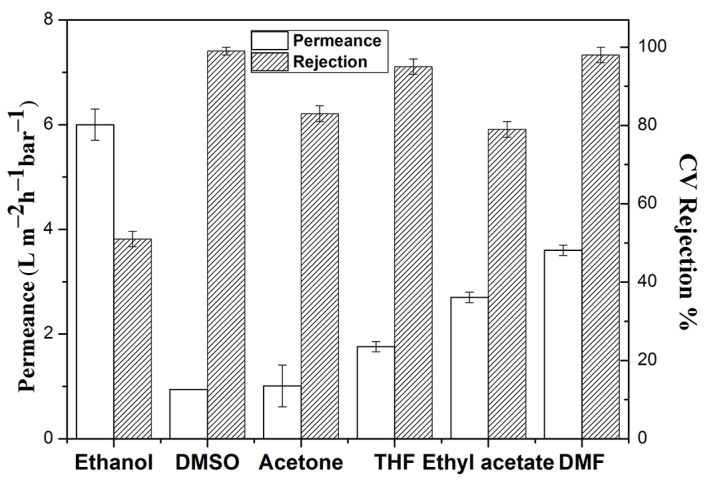
The performance of NF-E-2LWP membrane in ethanol, DMSO, acetone, THF, ethyl acetate and DMF.

Figure 8 displays the separation performance of the NF-E-2LWP membrane in several organic solvents, including ethanol, dimethyl sulfoxide (DMSO), acetone, tetrahydrofuran (THF), ethyl acetate, and DMF. The membrane exhibits high rejection rates of 95%, 99% and 98% in THF, DMF, and DMSO, respectively. The membrane has high permeability in ethanol, but its rejection rate for CV is relatively low. The separation performance of membranes in different solvents is closely related to the properties of the solvents. Factors such as solvent polarity, viscosity, and density lead to different interrelationships between membranes and solvents [36].

**Figure 9:**
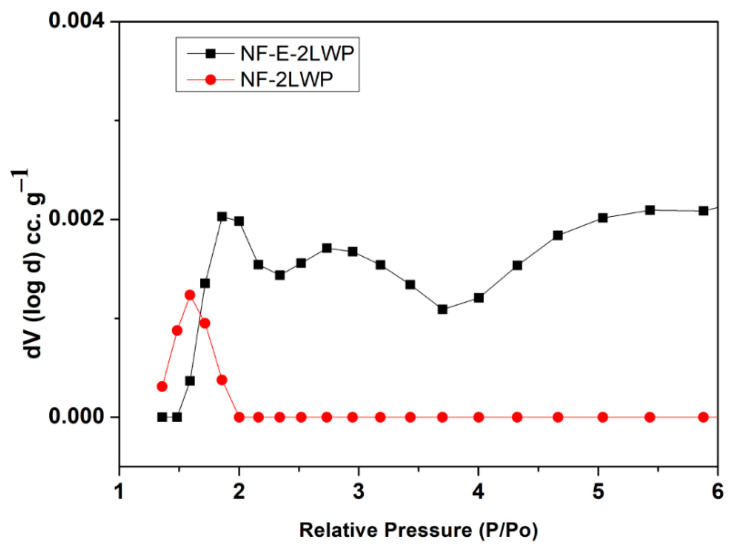
The pore size distribution of NF-2LWP and NF-E-2LWP as determined by means of the nitrogen adsorption and desorption method.

Furthermore, the pore size distribution was determined via the nitrogen adsorption and desorption method and is plotted in Figure 9. NF-E-2LWP has a wider pore size distribution than NF-2LWP, indicating that the modification of EDA increases more pore structures due to cross-linking.

**Figure 10:**
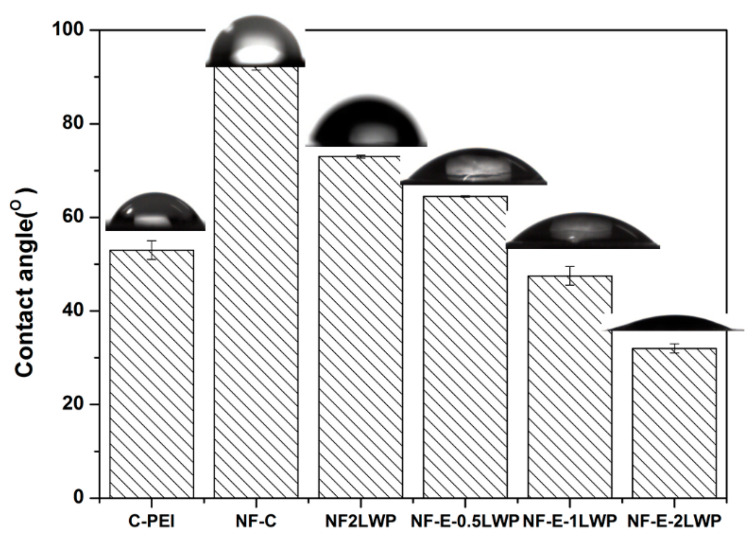
The static water contact angles of C-PEI, NF-C, NF-2LWP, NF-E-0.5LWP, NF-E-1LWP, and NF-E-2LWP membranes.

As shown in Figure 10, the C-PEI membrane has a smaller contact angle of 53° due to the presence of more amine groups. NF-C has a larger contact angle of 92.5° due to excessive TMC on the membrane surface, while the unmodified NF-2LWP has a contact angle of 73°. For EDA-modified membranes, the contact angle is generally smaller than that of unmodified ones. In addition, as the amount of LWP added increases, the contact angle of the membrane gradually decreases in size. NF-E-2LWP has a contact angle of 32°, which may be due to the influence of hydrophilic hydroxyl and amine groups.

**Figure 11:**
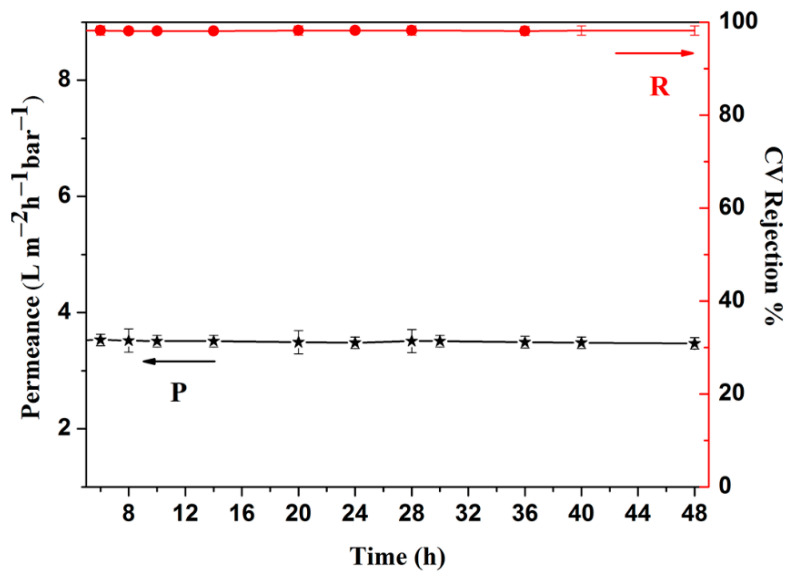
The performance of NF-E-2LWP when separating CV from DMF for 48 h, where the red trend line represents rejection and the black trend line represents permeance.

The long-term stability experiment of NF-E-2LWP is shown in Figure 11. After running the experiment in DMF for 48 h, the membrane rejection remained around 98% and the permeance remained around 3.6 L m^−2^ h^−1^ bar^−1^, demonstrating the potential of the membrane to operate in DMF.

Table 2 lists the separation performance of some polymeric membranes in an organic solution. For instance, Vankelecom et al. [37] prepared crosslinked PVDF membranes, showing DMF permeance of about 2.16 L m^−2^ h^−1^ bar^−1^ with an RB rejection rate of 75.0%. Li et al. [38] fabricated an OSN membrane based on pyromellitic dianhydride, 4′-diaminodiphenylmethane, which has DMF permeance of about 6.1 L m^−2^ h^−1^ bar^−1^ with an RB rejection rate of 92%. The synthesized NF-E-1LWP and NF-E-2LWP membranes are comparable to the above membranes in DMF.

## 4. Conclusions

We synthesized a liquefied walnut shell polyacrylate (LWP) membrane modified with ethylenediamine and discussed its separation performance and potential application in DMF recovery. The prepared OSN membrane maintained a stable DMF permeance of 3.53 L m^−2^ h^−1^ bar^−1^ and a rejection rate of 98% for crystal violet (CV, 407.98 g mol^−1^). The characterization via ATR-FTIR and Raman spectroscopy confirmed the successful modification of liquefied walnut shells with EDA. The load on the membrane is in the order of NF-E-2LWP > NF-2LWP > C-PEI membrane, which indicates that the mechanical properties of the modified membranes have also been greatly improved. The optimal membrane NF-E-2LWP has a contact angle of 32°, which may be due to the influence of hydrophilic hydroxyl and amine groups. The separation performance of the optimal membrane NF-E-2LWP in several organic solvents, such as ethanol, dimethyl sulfoxide (DMSO), acetone, tetrahydrofuran (THF), ethyl acetate, and DMF, showed high rejection rates of 95%, 99% and 98%, respectively. This study provides a useful reference for the preparation of solvent-resistant membrane materials with a high throughput and a high rejection rate using biomass.

## Figures and Tables

**Table 1:** The Poisson’s ratios of the C-PEI, NF-2LWP and NF-E-2LWP membranes.

Membrane	Test Conditions			
	Width (mm)	Thickness (mm)	Velocity (mm/min)	Poisson’s Ratio
C-PEI	11.08	0.24	1	0.38121
NF-2LWP	10.58	0.21	1	0.46513
NF-E-2LWP	10.72	0.25	1	0.458808

**Table 2:** A comparison of separation performance in DMF using different polymer membranes.

OSN Membrane	Solvent Permeance (L m^−2^ h^−1^ bar^−1^)		Solute	Mw of the Solute (g mol^−1^)	Rejection (%)	Ref.
Crosslinked PVDF	DMF	2.16	Rose Bengal	1017	75	[37]
Lenzing P84	DMF	1.67	Styrene oligomer	200	93	[39]
PMDA-ODA Polyimide	DMF	2.5	Rose Bengal	1017	96	[40]
PMDA-MDA Polyimide	DMF	6.1	Rose Bengal	1017	92	[38]
NF-E-1LWP	DMF	3.70	Crystal Violet	407	96	This work
NF-E-2LWP	DMF	3.53	Crystal Violet	407	98	

## Data Availability

All the data are available within the manuscript.

## Supplementary Materials

The following supporting information can be downloaded at: https://www.mdpi.com/article/10.3390/membranes13080719/s1, Table S1: The C, N and O compositions of the C-PEI, NF-C, NF-1NP, NF-2LWP and NF-E-2LWP membranes.
