# Supplementary material for: Preparation of a Solvent-Resistant Nanofiltration Membrane of Liquefied Walnut Shell Modified by Ethylenediamine
Source: Membranes (Basel). 2023 Aug 4;13(8):719. doi: 10.3390/membranes13080719 (PMC10456442; doi:10.3390/membranes13080719)
Supplement: Supplementary file 1 [file membranes-13-00719-s001.zip › membranes-2532166-supplementary.pdf]

Table S1. The C, N and O compositions and O/N valuess of the C-PEI, NF-C, NF-1NP, NF-2LWP and NF-E-2LWP membranes.

Membrane	Element content (atom. %)				
	C	N	O	S	O/N
C-PEI	76.00	8.45	14.75	0.20	0.194
NF-C	63.2	21.95	13.9	0.94	0.633
NF-2LWP	75.97	10.75	12.85	0.43	1.195
NF-E-1LWP	63.89	21.70	14.05	0.36	0.647
NF-E-2LWP	64.96	20.56	14.25	0.23	0.69
